# Optic neuritis and mydriasis after vaccination: a case report

**DOI:** 10.1186/s13256-024-04526-y

**Published:** 2024-04-25

**Authors:** Elcio Luiz Bonamigo, Pamela Kuyava, Taísa Sacomori Paula Zanotto Reginatto, Arthur Gabriel Duran, Luisa Truculo, Eglas Emanuel Rossi, Ricardo Alexandre Stock, Claudia Elisa Grasel

**Affiliations:** 1 University of West Santa Catarina, Street Getúlio Vargas, 2125, Neighborhood Flor da Serra, Street 13 de Maio, 314, Room 21, Joaçaba, Santa Catarina 89600-000 Brazil; 2 Unochapecó. Street Senador Atílio Fontana, 591-E, Neighborhood Efapi, Chapecó, Santa Catarina 89600-000 Brazil

**Keywords:** Optic neuritis, Mydriasis, Vaccines, Adverse effects, Case report

## Abstract

**Background:**

Optic neuritis (ON) is an inflammatory demyelinating condition of the optic nerve, with various causes. Its incidence is higher in children and young adults than in older adults of both genders, but is more common in women than in men. ON is rarely associated with mydriasis, and it is seldom triggered by vaccines against tetanus and diphtheria.

**Case report:**

A 36-year-old Caucasian woman presented with bilateral ON that had started 18 days after administration of a booster dose of the double adult vaccine (dT) against diphtheria and tetanus. Bilateral mydriasis persisted after treatment and clinical resolution of the ON. She experienced severe headache, blurred vision, decreased visual acuity in the right eye and bilateral mydriasis, a diagnosis confirmed by imaging tests. Treatment with oral corticosteroids resulted in rapid resolution of the neuritis; however, mydriasis persisted for several months.

**Conclusion:**

This study describes a very unusual case of bilateral ON associated with prolonged mydriasis after vaccination against tetanus and diphtheria that regressed after treatment with oral corticosteroids. Prolonged mydriasis was the manifestation that differed from the other cases previously described.

## Background

Vaccines are effective in preventing many diseases, but they are not free of side effects. Adverse events occurring between 2 and 30 days after vaccination are regarded as possible consequences of immunization [1]. Moreover, it is necessary to notify the Health Department of the respective municipality of these adverse events [2].

Vaccines against diphtheria and tetanus can be categorized as infant-type (DT) and adult-type, with the latter administered as a primary immunization or a booster dose. Adult type consists of diphtheria and tetanus toxoids as the main substances, aluminum hydroxide or phosphate as an adjuvant and thimerosal as a preservative, all packaged in an injectable vial.

Optic neuritis (ON) is a primary inflammation of the optic nerve, accompanied by demyelination of the nerve and associated axonal damage, that can lead to ganglionic loss and visual dysfunction [3]. ON may spare the optic disc (retrobulbar neuritis) or cause edema (papilitis) [4]. Worldwide, ON has an annual incidence of 0.94 to 2.18 per 100,000 persons and is due to various causes [5]. ON can occur at any age and in both sexes, being more common in children and young adults than in older adults and in women more than in men [6]. ON is one of the most serious adverse ophthalmologic reactions in response to vaccination, including the vaccine against diphtheria and tetanus, along with uveitis and retinal inflammation [7]. The mechanism by which a vaccine triggers ON has not yet been determined [8–10].

Mydriasis is a rare neurological symptom that may be indicative of nerve damage, but is very rarely associated with ON and its occurrence in the prolonged form has not yet been described [11]. Mean ± standard deviation pupil sizes among emmetropic presbyopes under conditions of low, medium, and high illumination have been reported to be 5.49 ± 0.94 mm, 3.37 ± 0.49 mm and 2.67 ± 0.34 mm, respectively [12]. However, although the patient's pupil was not paralytic, its diameter remained larger for several months.

This case report describes a patient treated at a private clinic in a city in the Midwest of Santa Catarina, in southern Brazil. This was a descriptive, retrospective and observational study, involving direct collection of data from medical records, complementary examinations and face-to-face interviews.

## Case presentation

A white woman aged 36 years, employee of a meat industry, presented at the office of a private ophthalmologist in a city in the Midwest of Santa Catarina. She was experiencing acute symptoms of bilateral visual blurring; reduced visual acuity, which was more prominent in the right eye; the sensation of a film in front of her eyes and severe headache, which started 4 days prior to presentation. She denied previous episodes or trauma and denied using anticholinergic substances. She reported that she had received a booster dose of the double adult vaccine against diphtheria and tetanus in the arm three weeks before. Four days later, she developed inguinal ganglia, which resolved spontaneously. Her visual symptoms started 18 days after immunization.

Physical examination showed total paralytic mydriasis in her right eye (RE), estimated at 8 mm, and total mydriasis with partial preservation of the pupillary photomotor reflex in her left eye (LE), also estimated at 8 mm, without changes in extrinsic eye movements or bilateral eyelid positioning. Visual acuity was 20/150 with spherical − 0.50 in her RE; and 20/20 with cylinder − 0.25 × 15 in her LE. In the near reading table (Jaeger table), she reached J1 (normal vision) with 1.75 addition. Intraocular pressure was 14 mmHg in the RE and 13 mmHg in the LE, with no other changes.

Laboratory tests showed that she was negative for HIV 1 and 2, syphilis, toxoplasmosis, and Covid-19, and that she was negative on a tuberculin test. Complete blood count, fasting blood glucose, antinuclear factor, rheumatoid factor, and human leukocyte antigen B27 were also tested, and urinalysis was performed.

The day after the consultation, the patient required evaluation at the local emergency room due to severe headache. A neurological investigation showed no alterations in her level of consciousness, reflexes, balance or sensitivity. Tomographic examination of the skull, performed without contrast, useful for investigating some causes of third nerve palsy such as tumors, showed results within normal parameters, and she was therefore discharged. The magnetic resonance imaging, that would have been useful to investigate multiple myelosis especially, was not performed by the neurologist. On the same day, she returned to the office, where she started treatment with oral prednisone, at a dose of 60 mg/day (1 mg/kg/day), for 10 days.

**Fig. 1:**
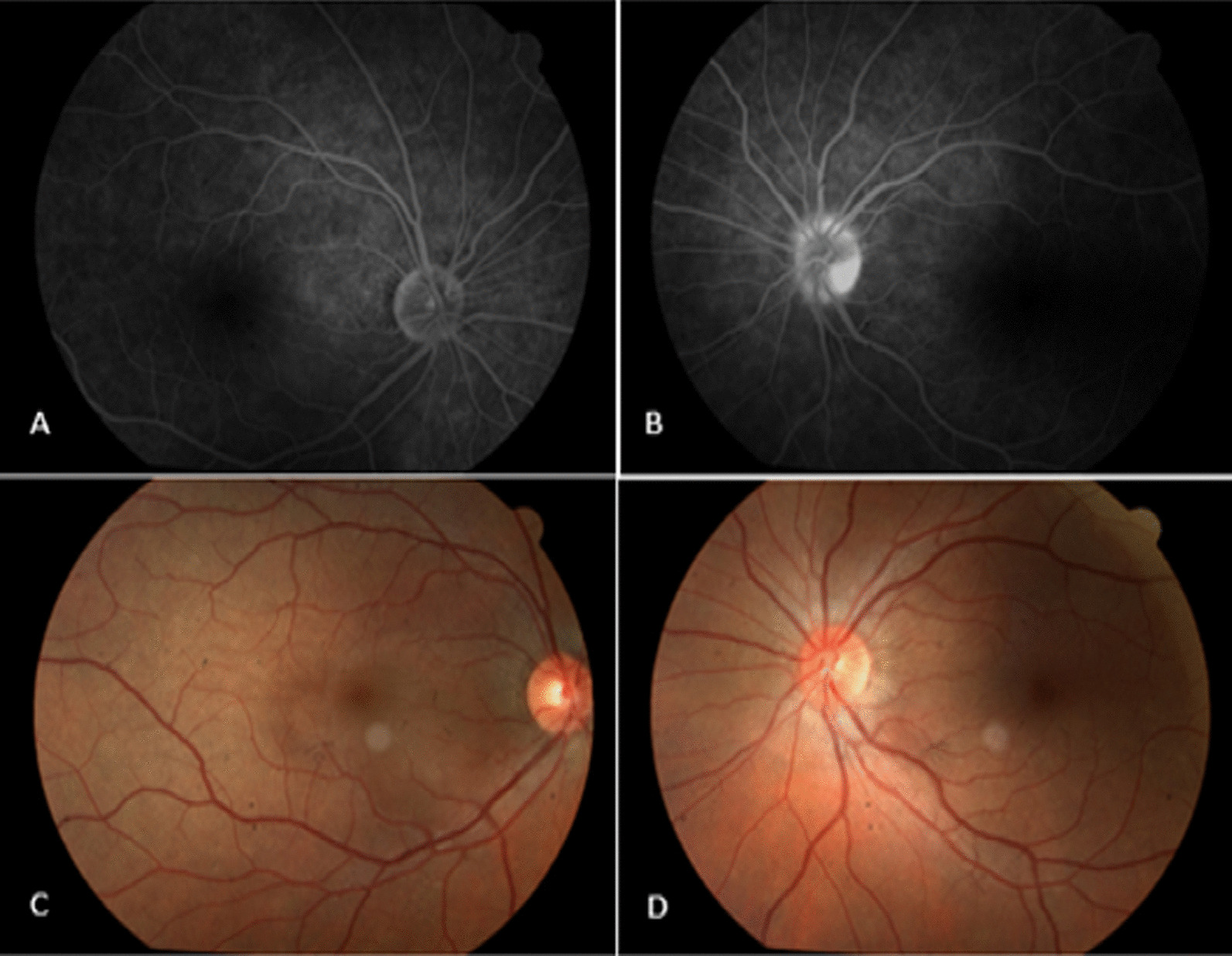
Results of angiography and retinography examinations performed on May 4, 2021. **A** Angiography of the right eye, showing physiology within normal limits. **B** Angiography of the left eye, showing papilla with preserved perfusion, blurring of the papillary border, and small adjacent retinal edema (papillary edema). **C** Retinography of the right eye, showing normal findings. **D** Retinography of the left eye, showing late papillary hyperfluorescence, with extravasation of contrast (papillary edema)

**Fig. 2:**
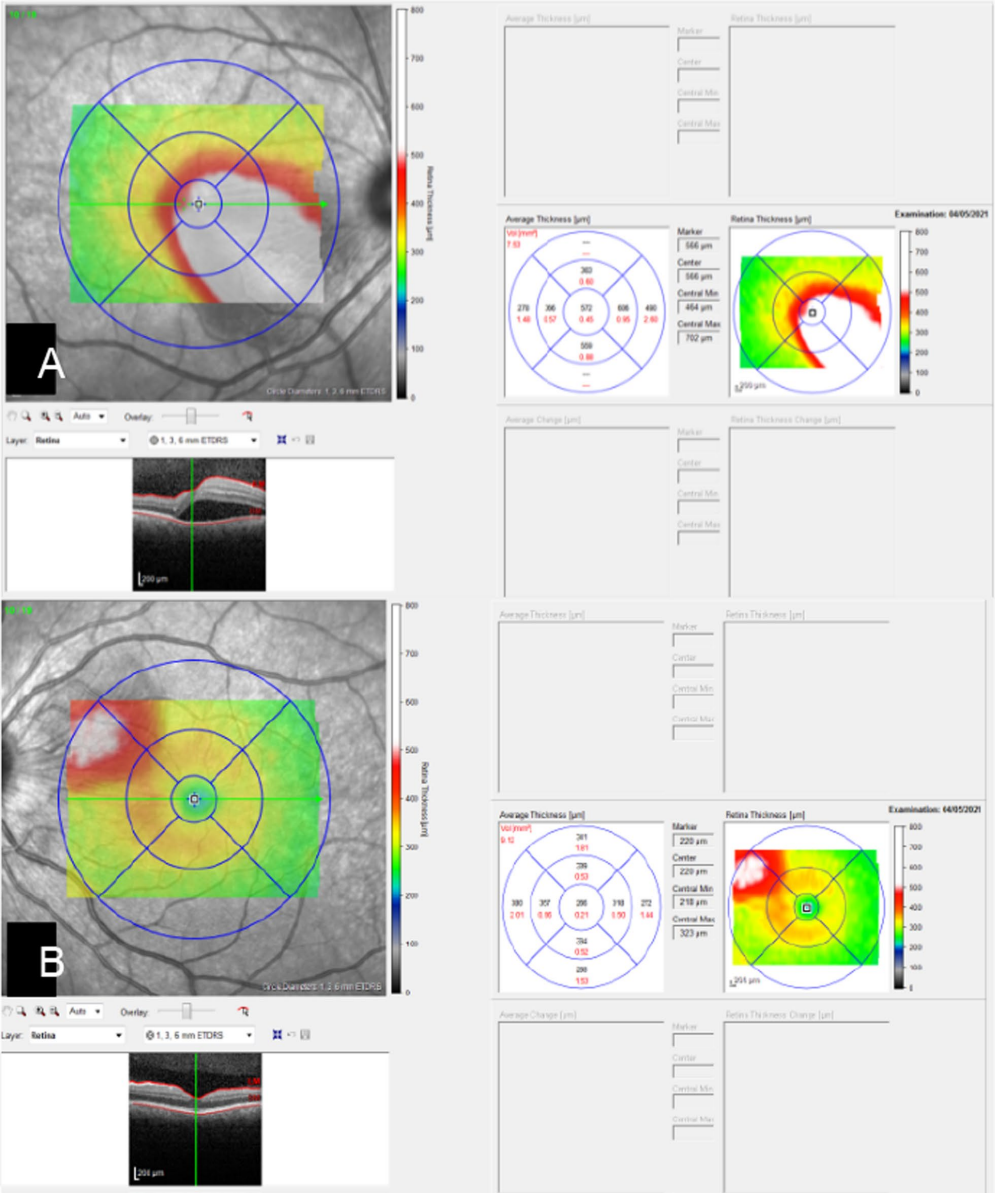
**A** Optical coherence tomography (OCT) of the papilla of the right eye, showing serous retinal detachment involving the foveal region. **B** OCT of the papilla of the left eye, showing peripapillary edema with a preserved foveal area

Retinography and digital angiography of the retinas showed ON in her left eye due to blurring of the papillary border, with a small adjacent retinal edema (papillary edema) and slight temporal pallor of the papilla. Optical coherence tomography (OCT) of the papillae showed optic neuritis in the RE, evidenced by serous retinal detachment peripapillary reaching the foveal region (Figs. 1 and 2). The lack of involvement of the choroid motivated the adoption of the diagnosis of optic neuritis.

**Fig. 3:**
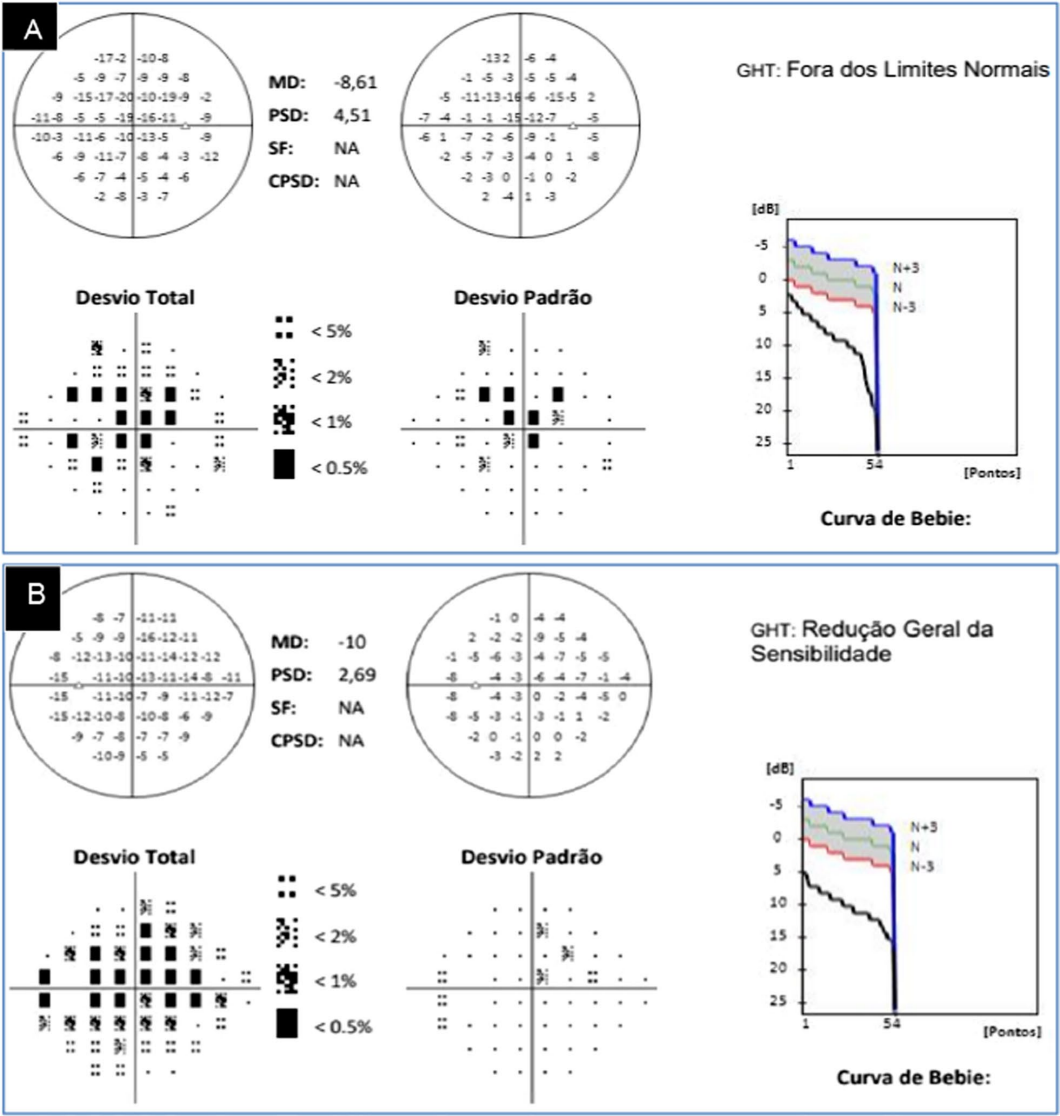
Results of computerized perimetry examinations performed on May 10, 2021. **A** Computerized perimetry of the right eye, showing central scotomas, a general reduction in sensitivity (mean deviation − 8.61) and glaucoma hemifield test (GHT) results outside normal limits. **B** Computerized perimetry of the left eye, showing mild central scotomas, overall reduced sensitivity (mean deviation − 10) and GHT outside normal limits. Desvio total, Total deviation; Desvio padrão, Standard Deviation; Dentro dos Limies Normais, Within normal limits; Curva de Bebie, Bebie Curve; Pontos, Points

Visual acuity in her right and left eyes was 20/40 and 20/20, respectively, on day 9 and 20/25 and 20/20, respectively, on day 12. Up close she reached J1 in the Jaeger table with a 0.75 addition. A visual field examination performed on day 12, showed central scotomas in the RE and mild scotomas in the LE (Fig. 3A, B), with mean deviations on the global mean deviation index of − 8.61 in the RE and − 10 in the LE.

**Fig. 4:**
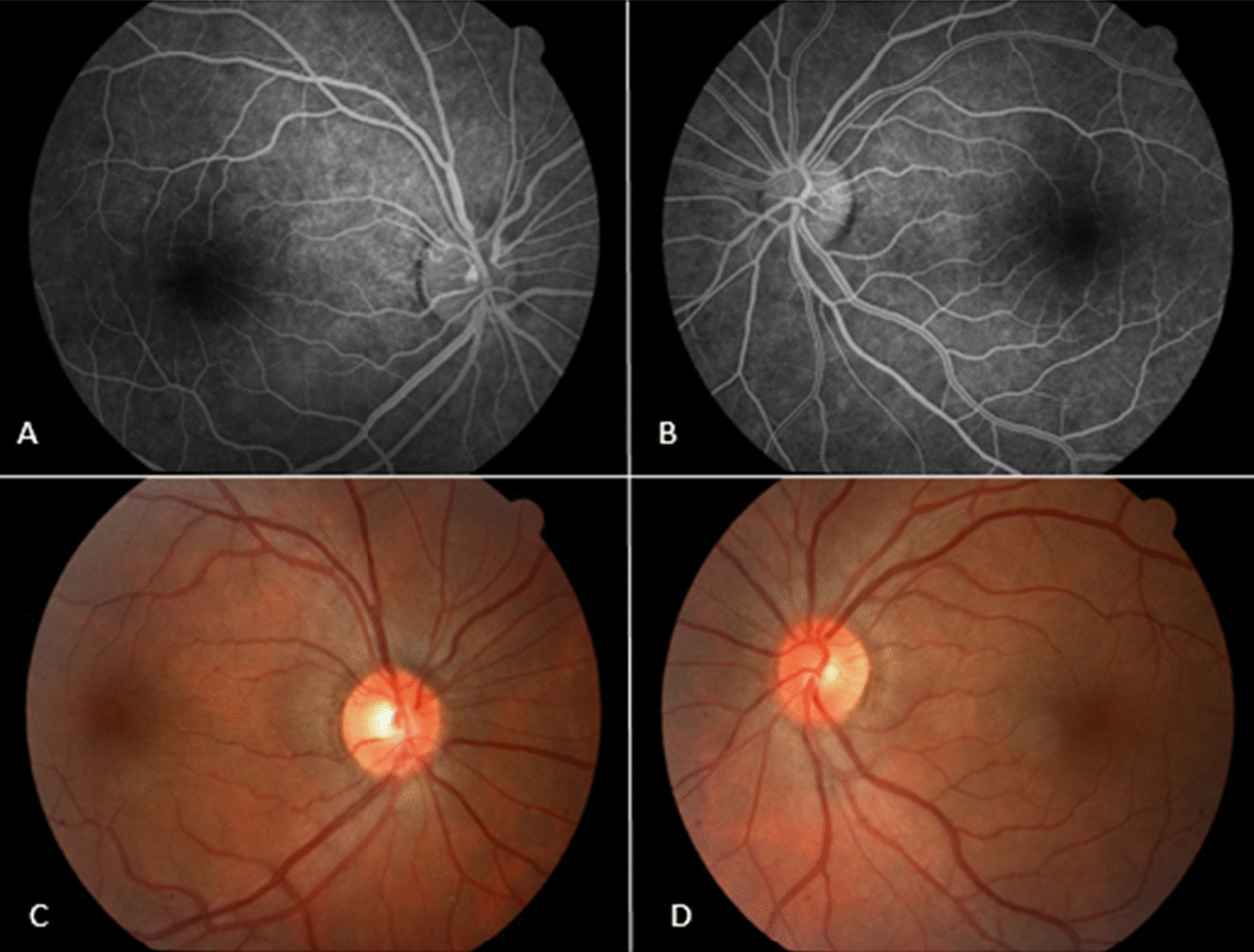
Results of angiography and retinography examinations performed on May 19, 2021. **A** Angiography of the right eye, showing physiological results within normal limits. **B** Angiography of the left eye, showing physiological results within normal limits. **C** Retinography of the right eye, showing results within normal limits. **D** Retinography of the left eye, showing results within normal limits

**Fig. 5:**
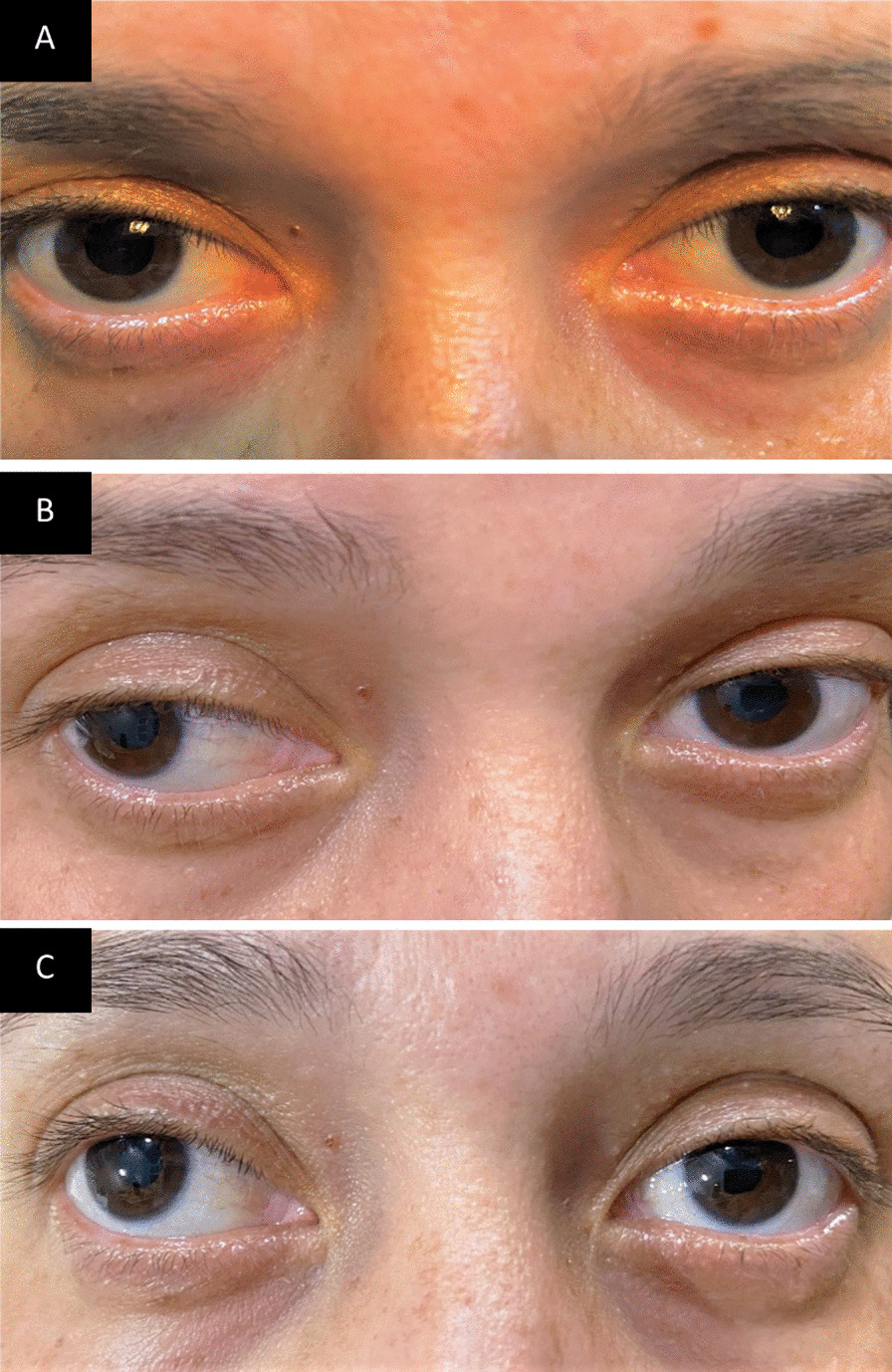
Photographs of the pupils. **A** Photograph of the pupils on May 10, 2021, 12 days after the onset of disease, showing bilateral mydriasis (5.5 mm). **B** Photograph of the pupils on July 10, 2021, 41 days after the onset of disease, showing a decrease in mydriasis (5 mm). **C** Photograph of the pupils on December 9, 2021, 8 months after the onset of the disease, showing a slight persistence of mydriasis, but without interference in accommodation (4.5 mm)

On the same day 12, she showed persistent bilateral mydriasis of approximately 5 mm (Fig. 5A). Repeat angiography of the retinas and retinography on day 21 showed normal results (Fig. 4). On day 41, she continued to show persistent mydriasis of 4.5 mm (Fig. 5B).

**Fig. 6:**
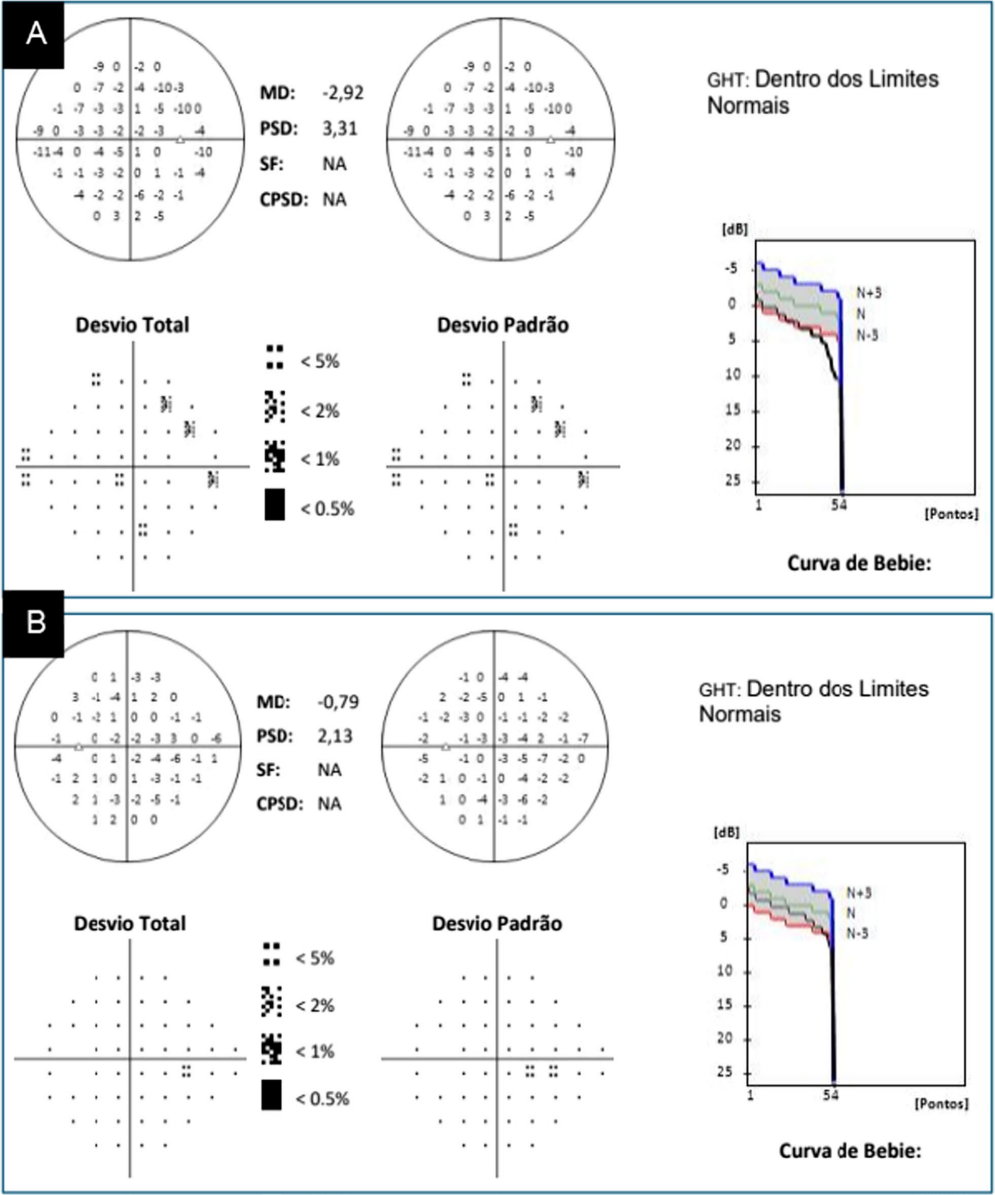
Results of computerized perimetry examinations performed on October 28, 2022. **A** Computerized perimetry of the right eye close to normal limits. **B** Computerized perimetry of the left eye within normal limits. Desvio total, Total deviation; Desvio padrão,  Standard Deviation; Dentro dos Limites Normais, Within normal limits; Curva de Bebie, Bebie Curve; Pontos, Points

Visual acuity in both eyes was 20/20 on days 133 and 180 (6 months). The patient continued to show persistent mild, bilateral mydriasis of 5 mm after 8 months (Fig. 5C). Although her visual acuity remained 20/20 in both eyes after 10 months, the patient experienced visual difficulty when straining. She was therefore prescribed refractive and spherical lenses, + 0.25 in the RE and flat degree in the LE. A visual field examination performed on October 26, 2022, 16 months and 10 days after the first consultation, showed superior temporal and nasal peripheral scotomas in the RE, but normal findings in the LE (Fig. 6A and B).

After 16 months, her visual acuity remained within normal parameters and she no longer felt the need to use refractive lenses. Table 1 shows the changes in this patient's visual acuity over the 16 months of disease progression. Although the pupils were still slightly above the standards considered normal, both had normal photomotor reflexes and accommodation.

**Table 1:** Changes in the patient’s visual acuity over 16 months, with correction when necessary

	Visual acuity	
	Right eye	Left eye
1 day	20/150	20/20
9 days	20/40	20/20
12 days	20/25	20/20
133 days	20/20	20/20
6 months	20/20	20/20
10 months	20/20	20/20
16 months	20/20	20/20

## Discussion and conclusion

The present study describes a patient who developed bilateral ON, along with prolonged bilateral mydriasis, after a booster dose of the adult diphtheria and tetanus vaccine. ON has been defined as any inflammatory or demyelinating disease of the optic nerve resulting from an infectious process, an autoimmune condition or a disorder triggered by infectious agents, including vaccines [13]. Acute ON presents as an isolated clinical event without additional neurological involvement (monosymptomatic) [8]. Its primary clinical feature is rapid onset with loss of vision within 1 to 2 days, often accompanied by pain on eye movement, reduced brightness perception, colored vision, and/or headache [14]. The patient described in the current study presented with headache and visual impairment in the RE and difficulty in near vision in both eyes [1].

ON as an adverse effect of administration of a vaccine against diphtheria, tetanus, pertussis and poliomyelitis was first reported in a 27-year-old Hispanic man who presented to the emergency room with headache for 5 days, pain on eye movement and blurred vision that started one day after immunization [15]. The present patient developed bilateral inguinal nodes 4 days after vaccination, suggesting a causal link between the vaccine and the subsequent immunological reactions. ON as an adverse effect of vaccination is unilateral in 31% of patients and bilateral in 69% [9]. Neuritis can be retrobulbar, with a normal optic disc, or undetectable by all diagnostic methods [4, 10]. In the present patient, ON of the RE was only evident on an OCT scan. Due to the lack of involvement of the choroid and involvement of only the juxta papillary retina, the denomination optic neuritis was adopted and not optic neuroretinitis.

The profile presented by the present patient agrees with previous findings. ON has been reported to be more frequent in younger than in older adults and to be more prevalent in women than in men, with a mean age in women of 30 years [4, 7, 8]. ON has been reported to affect 1–3 per 100,000 individuals per year [4, 7, 8]. ON usually occurs after a booster dose of vaccine, with a time delay between vaccination and symptoms ranging from 6 h to 21 days [9]. Symptom onset has also been reported to occur 2 to 30 days after vaccination, with an average of 8.2 days [16]. The present patient experienced ocular manifestations 18 days after vaccination, with inguinal nodes after 4 days.

This patient had symptoms common to those in patients with ON, such as headache and changes in visual acuity [14]. Decrease visual acuity only in the right eye, although not detected by angiography, is explained by changes in the OCT. This patient also developed mydriasis, an extremely rare event in patients with ON. Contact with products containing atropine (hyoscyamine), hyoscine (scopolamine), pirenzepine, telenzepine, tropicamide, cyclopentolate, benzatropine and ipratropium, or their derivatives, all of which may cause mydriasis, was ruled out [17].

The possibility of Adie-Holmes syndrome, also called Adie’s tonic pupil, was also excluded because it is a condition of bilateral mydriasis, which usually occurs after viral infection [18]. The possibility of Marcus Gunn’s pupil, which is caused by incomplete damage to the optic nerve, was also excluded. Also, especially due to the bilateral demonstration an orthophoria, third nerve palsy was ruled out. Mydriasis is rarely associated with ON, but is a rare neurological sign suggestive of optic nerve damage [11].

Although the risk of developing a demyelinating syndrome of the central nervous system after vaccination is quite low, estimated at 0.1%, it is not negligible [15]. ON triggered by an immune mechanism is the most frequent post-vaccination acute demyelinating syndrome [8]. This ON triggers a process of inflammatory demyelination [4]. Demyelination is a pathological process, in which normally myelinated nerve fibers lose their insulating myelin layer [10]. The presence of central scotomas on computerized perimetry is an indicator of the demyelinating condition.

Adjuvants have been shown to play a pathogenic role in the induction of autoimmune syndromes. The effectiveness of most vaccines depends on the presence of an adjuvant along with the infectious antigen. Currently, the most frequently used adjuvant is aluminum, which is present in double adult vaccine [8].

Effective treatment of ON includes the early administration of pulse or oral corticosteroids [6]. Although neuritis is also an adverse effect of COVID-19 vaccines, its severity is not considered minor [19]. The present patient reported tolerating the treatment well and being satisfied with the result, but she worried about losing her vision and had moments of great anxiety.

Skull computed tomography instead of magnetic resonance imaging was one limitation of the research, but the appearance of symptoms a few days after vaccination highlights the possibility of one association.

This report described a patient who developed bilateral ON, along with bilateral mydriasis, after a booster injection of a vaccine against diphtheria and tetanus. Her initial complaints were bilateral visual blurring, which was more prominent in the RE, a sensation of film in front of her eyes and severe headache. Treatment with oral corticosteroids, however, resulted in good visual recovery.

The development of ON after immunization with vaccine against diphtheria and tetanus is rare, as is the association of ON with mydriasis. Prolonged mydriasis was the manifestation that differed from the other cases previously described.

## Data Availability

The author’s original image files are available within the article.
